# Prevalence of Medical Credit Cards by Specialty

**DOI:** 10.1001/jamahealthforum.2025.0174

**Published:** 2025-04-11

**Authors:** Joseph Dov Bruch, Cal Chengqi Fang, Betsy Q. Cliff

**Affiliations:** 1 Department of Public Health Sciences, The University of Chicago, Chicago, Illinois

## Abstract

This cross-sectional study assesses which medical specialties are more likely to contract with financial firms to offer medical credit cards.

## Introduction

Financial institutions are increasingly marketing medical credit cards as a solution to rising medical debt.^1^ Medical credit cards offer deferred interest terms, allowing patients to avoid interest payments for a promotional period of 6 to 18 months. However, if the balance remains unpaid by the end of this period, accrued interest from the start date is added to the balance.^2^ While the promotional period has the potential to allow relatively frictionless borrowing and alleviate financial burdens for patients, the average annual percentage rate on medical credit cards is 26.99%, which tends to be higher than other payment types. A recent survey found that about a quarter of patients who financed through medical credit cards did not pay off the balance in time to avoid the deferred interest.^3^ Medical credit cards were used to pay $23 billion in health care expenses, resulting in $1 billion in deferred interest payments from 2018 to 2020.^2^

There are 3 major medical credit cards, each sold by financial institutions or their subsidiaries: Alphaeon (Comenity Capital Bank of Bread Financial), CareCredit (Synchrony Financial), and Wells Fargo Health Advantage (Wells Fargo).^2^ Physician practices contract with these financial institutions—often receiving software, training, and promotional material for medical credit cards in exchange for paying a processing fee and agreeing to accept the card as payment.^4^ It is unknown which types of specialties are more likely to offer these products.

## Methods

Alphaeon, CareCredit, and Wells Fargo have search features on their websites that allow patients to find physician practice locations where these products are accepted (contracted) using a zip code or an address. We used web-scraping techniques to identify all practice locations on these websites (eMethods in Supplement 1). The process involved querying the websites using a zip code list obtained from the US Census Bureau, covering 42 724 zip codes. We extracted data, including the address and medical specialty, then cleaned the data by removing duplicates and standardizing specialty descriptions. The final dataset comprised 180 311 unique locations. Using estimates derived from OneKey^5^ and the Census Bureau^6^ on the total number of physician practice locations by specialty, we calculated for each specialty the number and percentage of practice locations that contracted with a medical credit card firm.

This study did not include human participants and was not subject to an institutional review board in accordance with 45 CFR §46. We followed the STROBE reporting guideline for cross-sectional studies.

## Results

Of the 180 311 identified practice locations, 67.0% of dentistry, 45.7% of podiatry, 29.7% of chiropractic, 25.5% of physical medicine and rehabilitation, 20.5% of dermatology, 18.3% of pharmacy, 14.0% of imaging and radiology, and 8.7% of orthopedic surgery contracted with financial firms to offer medical credit cards (Table). These practice locations exist across the country, particularly in the Northeast (Figure).

**Table.** Medical Credit Card Prevalence by Specialty

Specialty	Practice locations accepting medical credit cards	Total practice locations^a^	%
Dentistry	90 644	135 333	67.0
Podiatry	3474	7597	45.7
Chiropractic	11 908	40 118	29.7
Physical medicine and rehabilitation	2913	11 437	25.5
Dermatology	2728	13341	20.5
Pharmacy	8029	43879	18.3
Imaging and radiology	1666	11914	14.0
Orthopedic surgery	1594	18 366	8.7
Obstetrics and gynecology	1564	23 916	6.5
Urology	580	10 428	5.6
Anesthesiology	499	13 428	3.7
Family and general practice	2499	73 745	3.4
Neurology	297	11 528	2.6
Gastroenterology	341	13 395	2.6
Occupational therapy	168	18 366	0.9
Internal medicine	469	60 238	0.8
Pediatrics	201	29 452	0.7
General surgery	116	21 191	0.5
Medical laboratory	49	12 663	0.4
Emergency and critical care	30	15 646	0.2
Vision medicine	22 681	NA^b^	NA
Cosmetic medicine	15 590	NA^b^	NA
Outpatient surgery	1777	NA^b^	NA
Behavioral health	1133	NA^b^	NA
Vascular surgery	733	NA^b^	NA
Urgent care/walk-in clinics	498	NA^b^	NA
Sleep medicine	420	NA^b^	NA
Otolaryngology	414	NA^b^	NA
Immunology	290	NA^b^	NA
Speech therapy	112	NA^b^	NA
Other^c^	273	NA^b^	NA
Total	180 311^d^	NA	NA

Abbreviation: NA, not applicable.

^a^ The numbers of dentistry, pharmacy, podiatry, and medical laboratory practice locations were obtained from the 2021 County Business Patterns data released by the US Census Bureau. All other specialty practice locations were obtained from the 2023 IQVIA US physician specialties market insights report.

^b^ A reliable source estimate of total practice locations was unavailable.

^c^ The other category includes acupuncture, rheumatology, endocrinology, nephrology, nutritionists and dieticians, home health care, and colorectal surgery/proctology. These specialties were grouped together owing to small sample sizes.

^d^ The summation of all practice locations does not equate to the total because some practice locations provide more than 1 specialty.

**Figure.**
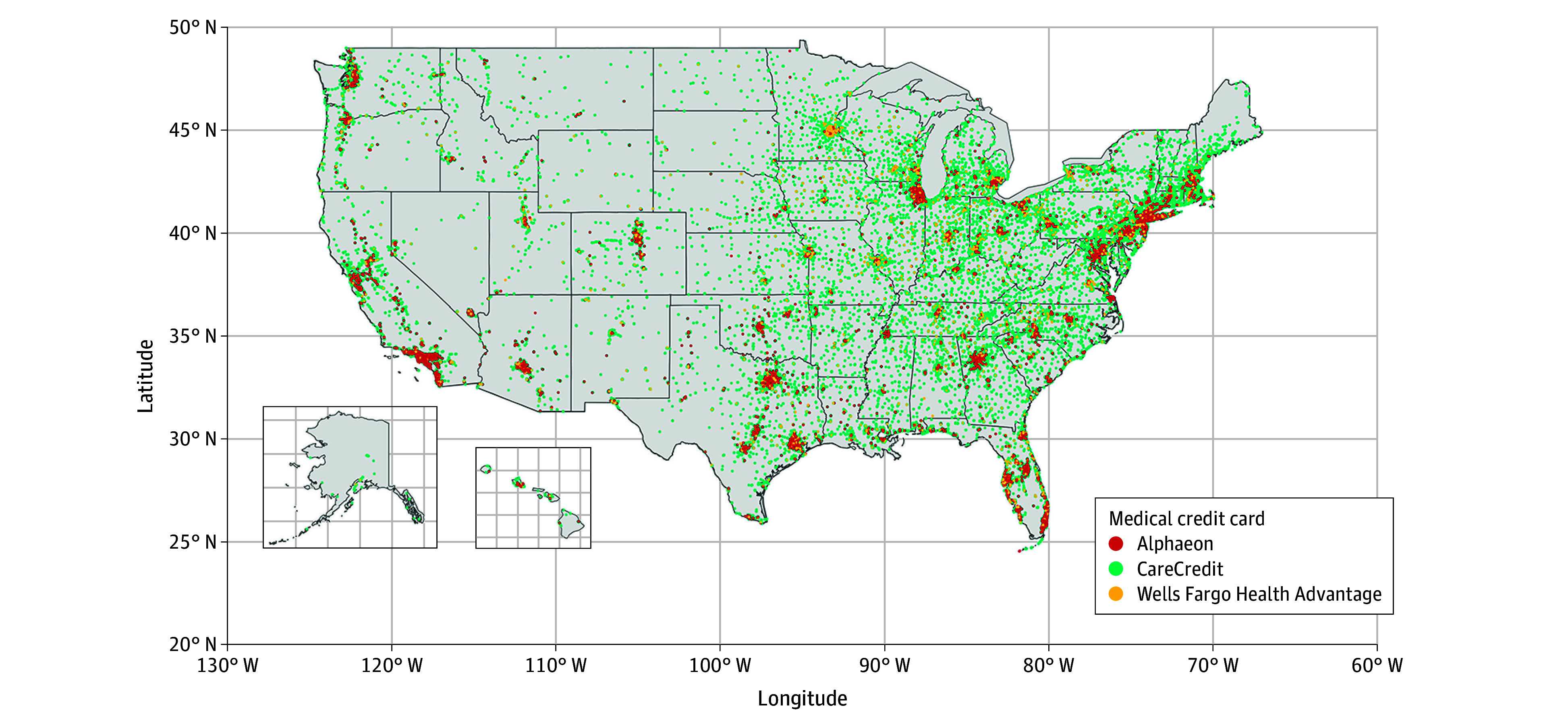
Medical Practice Locations Accepting Medical Credit Cards Each dot represents a medical practice location that accepts 1 of the 3 types of medical credit cards. The color of each dot indicates the specific medical credit card accepted at the location. A total of 180 311 unique practice addresses were obtained using each credit card’s locator service through web scraping (eMethods in Supplement 1) and geocoded using the Google Maps Geocoding API.

## Discussion

This cross-sectional study showed that more than 180 000 practice locations contracted with financial institutions to offer medical credit cards. While payment plans, low-interest installment loans, and other financing options tend to offer more generous and forgiving terms for patients unable to pay in full, some practices may lack the financial resources to offer these options. Medical credit cards may be appealing to physician practices because they ensure that the practices receive the full payment for services without enduring the burdens associated with bill collection.^2^ That said, patients with low financial literacy may have a hard time understanding the terms of the cards and may be vulnerable to deferred interest payments after the promotional period.

While specialties with the highest offer rates provide services less likely to be covered by insurance, we were unable to identify specific services associated with medical credit card use. We were also unable to identify which patients use these cards or whether practices have alternative financing options. As medical debt continues to rise, the impact of medical credit cards on patients’ finances and health should be an area of active research.

## Notes

Supplement 1.
eMethods

Supplement 2.
Data Sharing Statement
