# Supplementary material for: Prevalence of Medical Credit Cards by Specialty
Source: JAMA Health Forum. 2025 Apr 11;6(4):e250174. doi: 10.1001/jamahealthforum.2025.0174 (PMC11992600; doi:10.1001/jamahealthforum.2025.0174)
Supplement: Supplement 1. — eMethods [file jamahealthforum-e250174-s001.pdf]

Supplemental Online Content

Bruch JD, Fang CC, Cliff BQ. Prevalence of medical credit cards by specialty. *JAMA Health Forum*. 2025;6(4):e250174. doi:10.1001/jamahealthforum.2025.0174

eMethods

This supplemental material has been provided by the authors to give readers additional information about their work.

© 2025 Bruch JD et al. *JAMA Health Forum*.

eMethods.

Scraping, Cleaning, and Regrouping Practice Locations

Retrieving health facilities that receive medical credit cards by zip codes

None of the three medical credit card issuers have a publicly available list of health facilities that accept their credit cards. Instead, they provide locators on their websites that generate a list of health facilities accepting the credit cards when specific zip codes and/or specialties are input.

Medical Credit Card Issuer	***Locator***
CareCredit credit card (Synchrony Financial)	https://www.carecredit.com/find-a-location/
Wells Fargo Health Advantage card (Wells Fargo)<br>Note: Wells Fargo is no longer accepting credit applications under the Wells Fargo Health Advantage program but will continue to support existing Wells Fargo Health Advantage cardholders through September 2025.	https://retailservices.wellsfargo.com/locator/get-started
Alphaeon credit card (Comenity Capital Bank of Bread Financial)	https://goalphaeon.com/doctor-locator

We utilized web scraping techniques to obtain information about health facilities that contracted with these three medical credit card issuers. Specifically, we obtained a list of all US zip codes from the data.census.gov website using the uszipcode python module. Then, we created one script for each medical credit card that iterated over a list of 42,724 zip codes. Our scripts sent each zip code to the locators, retrieved the responses, and extracted relevant information such as the name, address, phone number, and specialties of healthcare providers from the responses. Due to the ranking algorithm's consideration of relevance and popularity, the retrieved results sometimes included locations with different zip codes from the one sent. The script saved only those results with matching zip codes and continued loading the "next page" until five consecutive locations had mismatched zip codes. The script was configured to load a maximum of 70 pages per zip code. This allowed us to recover approximately 80% of all practice locations that the issuers claim to serve. We followed web scraping best practices based on previous studies.[1,2]

These steps were conducted using Python 3.11.5. CareCredit credit card locator was scrapped on December 18, 2023; Wells Fargo Health Advantage card locator was scrapped on December 19, 2023; and Alphaeon credit card locator was scraped on December 31, 2023.

© 2025 Bruch JD et al. *JAMA Health Forum*.

Cleaning the health facilities' information

The data scraped from the three medical credit card issuers' websites contained duplicates and had different categorizations of specialties. For our analysis, we dropped duplicates created during the scraping process and renamed columns for consistency. We standardized the address and city fields and removed additional duplicates based on address and phone number. After this step, we had 209,138 unique health facilities from CareCredit, 3,383 from Wells Fargo Health Advantage, and 7,961 from Alphaeon credit card (Comenity Capital Bank of Bread Financial).

Entries related to veterinary and animal practices were removed from the CareCredit and Alphaeon datasets, as these services were not of interest. Descriptions that contain multiple specialties in the CareCredit and Alphaeon datasets were split into multiple rows, with each row representing a single specialty. We cleaned specialty descriptions by removing unnecessary white spaces and newline characters, ensuring that each facility providing multiple specialty services had one row per specialty. Certain specialty categories were renamed for consistency, and a comprehensive list of specialties was created, grouping them into broader categories (e.g., dentistry, vision medicine, and gastroenterology). Categories that are not considered medical specialties (e.g., medical equipment) were filtered out.

The cleaned datasets from the three providers were then merged, and duplicates caused by regrouping specialties were removed. Finally, practices located in US territories were filtered out, retaining only those in the 50 states and DC.

These steps were conducted using R 4.3.1.

eReferences

1. Ahmed A, Li X. Labor Unionization Among Physicians in Training. *JAMA*. 2023;330(19):1905-1906. doi:10.1001/jama.2023.17494

2. Cai C, Hone T, Millett C. The heterogeneous effects of China's hierarchical medical system reforms on health service utilisation and health outcomes among elderly populations: a longitudinal quasi-experimental study. *Lancet*. 2023;402 Suppl 1:S30. doi:10.1016/S0140-6736(23)02141-4

© 2025 Bruch JD et al. *JAMA Health Forum*.
