# Supplementary material for: Prevalence of Medical Credit Cards by Specialty
Source: JAMA Health Forum. 2025 Apr 11;6(4):e250174. doi: 10.1001/jamahealthforum.2025.0174 (PMC11992600; doi:10.1001/jamahealthforum.2025.0174)
Supplement: Supplement 2. — Data Sharing Statement [file jamahealthforum-e250174-s002.pdf]

Data Sharing Statement

Bruch. Prevalence of Medical Credit Cards by Specialty. *JAMA Health Forum*. Published April 11, 2025. doi:10.1001/jamahealthforum.2025.0174

Data
Data available: Yes
Data types: Data (not involving human participants)
How to access data: Data requests can be sent to Joseph Dov Bruch; jbruch@bsd.uchicago.edu
When available: With publication

Supporting Documents
Document types: None

Additional Information
Who can access the data: anyone requesting the data
Types of analyses: for any purppose
Mechanisms of data availability: without investigator support
